# Development of *Phyllanthus emblica* Extract-Loaded Niosomes for Cancer Treatment: Formulation and In Vitro Evaluation

**DOI:** 10.3390/ph19040582

**Published:** 2026-04-06

**Authors:** Al-Zahraa Khalifa, Naglaa Gamil Shehab, Dema Layth Jabbar, Heba Marwan Ibrahim, Manar Ahmed Hawash, Maryam Jamal Afif Said, Aliasgar Shahiwala, Bazigha K. Abdul Rasool

**Affiliations:** 1 Pharmaceutical Sciences Department, College of Pharmacy, Dubai Medical University, Dubai 20170, United Arab Emirates; alzahraa@dmu.ae (A.-Z.K.); dr.asgar@dmu.ae (A.S.); 2 Pharmacognosy Department, Faculty of Pharmacy, Cairo University, Cairo 11562, Egypt; 3 College of Pharmacy, Dubai Medical University, Dubai 20170, United Arab Emirates; deema.laeth@gmail.com (D.L.J.); hebamarwanibrahim@gmail.com (H.M.I.); manarhawash555@gmail.com (M.A.H.); s_we_we@hotmail.com (M.J.A.S.)

**Keywords:** *Phyllanthus emblica*, amla extract, niosomes, polyphenols, anticancer activity, controlled drug delivery, breast cancer, colorectal cancer, molecular docking, in silico pharmacokinetics

## Abstract

*Phyllanthus emblica* (amla) exhibits anticancer activity, but its extracts often suffer from poor stability and bioavailability. This study developed amla extract-loaded niosomes to enhance delivery and evaluate their anticancer activity against MCF-7 and HCT116 cell lines, supported by in silico analyses. **Methodology:** Amla extract was prepared using a 50% aqueous–alcoholic solvent system and lyophilized. Niosomes were prepared by the thin-film hydration method and characterized for physicochemical properties. Anticancer activity was evaluated through in vitro cytotoxicity studies, supported by molecular docking and in silico pharmacokinetic analyses. **Results:** Optimized niosomes exhibited spherical morphology, good homogeneity (PDI < 0.30), anionic surface charge, high entrapment efficiency (70.5 ± 5.9%), and sustained diffusion-controlled release. In vitro cytotoxicity demonstrated a strong concentration-dependent anticancer activity of amla-loaded niosomes across a range of concentrations (31.25–1000 µg/mL) against both MCF-7 and HCT116 cell lines. At 1000 µg/mL, cell viability decreased to 7.0% and 5.4% in MCF-7 and HCT116 cells, respectively, with calculated IC_50_ values of 245 µg/mL and 158 µg/mL. Molecular docking and pharmacokinetic predictions supported the potential multi-target anticancer relevance of major phytochemicals, including hydrolyzable tannins, phenolic acids, flavonoid aglycones and glycosides, and highlighted bioavailability limitations for certain high-affinity glycosylated flavonoids, reinforcing the rationale for vesicular encapsulation. **Conclusions:** Amla extract-loaded niosomes represent a promising vesicular system for enhanced, sustained delivery of anticancer activity in vitro, with complementary in silico findings supporting mechanistic plausibility and translational rationale. Further studies are warranted to evaluate their performance in vivo.

## 1. Introduction

Cancer remains a major global health challenge and one of the leading causes of death worldwide. According to recent estimates, approximately 19.3 million new cancer cases and nearly 10 million deaths occurred globally in 2020, and this number is projected to rise significantly in the coming decades. Despite advances in diagnosis and treatment, current therapies often face limitations such as systemic toxicity and drug resistance, highlighting the need for safer and more effective therapeutic strategies [1].

Humans have thought about medicinal plants and their use in treating diseases since the dawn of recorded history. Numerous statistical studies conducted nowadays indicate that the usage of medicinal plants is expanding globally [2]. Several factors have increased the use of these plants, their extracts, and their essential oils worldwide, including their availability, fair pricing, fewer side effects than chemical medications, and low cost.

Recent research has proved the usefulness of using herbal remedies for disease prevention and therapy [2]. *Phyllanthus emblica* fruit (amla), family *Phyllanthaceae,* is one of the herbal plants with many important therapeutic uses. It is the most significant therapeutic plant in the Indian traditional medical system, often known as Indian gooseberry or amla [3]. It plays a role in improving memory, lowering cholesterol, neutralizing snake venom, and is used for infectious diseases as a potent immunomodulatory [4].

*Phyllanthus emblica* has been reported to exhibit promising anticancer potential in preclinical studies, largely attributed to its antioxidant and bioactive phytochemical constituents [5]. In addition, the fruits showed antioxidant, anti-inflammatory, antimutagenic, and immunomodulatory activity. In our earlier investigation, the phytochemical constituents of *Phyllanthus emblica* fruit extract were systematically characterized, confirming the presence of multiple bioactive compounds belonging to flavonoids, phenolic acids, and water-soluble vitamins [6]. These constituents are considered key contributors to the extract’s therapeutic potential. However, despite their promising biological activity, the effective application of such phytochemically complex extracts is often limited by poor stability, rapid metabolism, and suboptimal bioavailability.

Advanced drug delivery systems have emerged as a promising strategy to address these limitations by improving drug stability, bioavailability, and therapeutic efficacy [7]. Recent studies have demonstrated that advanced vesicular and nanostructured drug delivery systems can improve drug stability, pharmacokinetic behavior, and therapeutic efficacy of anticancer agents by enhancing controlled release and cellular uptake [8]. Niosomes are non-ionic surfactant-based vesicular systems first introduced in the 1970s as an alternative to liposomes for drug delivery. They are composed mainly of nonionic surfactants and cholesterol, forming bilayer vesicles that can encapsulate both hydrophilic and lipophilic drugs. Due to their biocompatibility, stability, low cost, and ability to enhance drug bioavailability and control drug release, niosomes have attracted considerable attention in pharmaceutical research. Numerous studies have demonstrated their potential to improve the therapeutic efficacy of anticancer agents and other bioactive compounds by enhancing cellular uptake and targeted delivery [9,10].

Recent studies have highlighted the growing role of advanced nanocarrier systems in improving the delivery, stability, and therapeutic efficacy of anticancer drugs. For example, nanoscale drug delivery platforms and prodrug nanoassemblies have demonstrated enhancement in drug release control, improved pharmacokinetic behavior, and superior antitumor activity compared with conventional formulations [11,12]. By modulating vesicle composition, size, and surface properties, niosomes can alter drug pharmacokinetics, enhance cellular uptake, and improve therapeutic efficacy [9,10,13].

In addition, in our previous study, the phytochemical profile of *Phyllanthus emblica* fruit extract was comprehensively characterized using chromatographic and spectroscopic techniques, revealing the presence of several bioactive constituents, including flavonoids, phenolic acids, and hydrolyzable tannins, which are known for their antioxidant and anticancer properties [6]. These phytochemicals are considered key contributors to the extract’s therapeutic potential.

Although *Phyllanthus emblica* nanoformulations are less extensively reported than some other botanicals, the available literature supports the use of vesicular and polymeric platforms to enhance delivery, provide nanoscale systems, and enable high extract loading with controlled release/permeation. For example, emblica-extract liposomes have been reported as nanoscale unilamellar vesicles with high entrapment efficiencies [14]. More recent work also describes *emblica* polyphenol liposomes with high encapsulation efficiency (~83%) and improved stability [15]. Liposomal *emblica* extract has additionally been incorporated into gels and evaluated using in vitro/ex vivo release/permeation designs, supporting improved delivery compared with non-liposomal controls [16]. Polymeric nanoparticle systems for *Emblica officinalis* extracts have likewise been characterized using core benchmarks, including entrapment efficiency and release testing. *Phyllanthus emblica* has also demonstrated promising cytotoxic activity and has been incorporated into silver nanoparticles to enhance therapeutic efficacy [17]. However, its application in niosomal drug delivery systems for anticancer therapy remains largely unexplored.

Within this context, the present study was designed to develop and optimize *Phyllanthus emblica* extract-loaded niosomes as a potential delivery system for phytochemical constituents with anticancer activity. The formulations were optimized and characterized in terms of their physicochemical properties, including vesicle size, polydispersity, zeta potential, entrapment efficiency, and in vitro release behavior. The anticancer activity of the optimized formulation was then evaluated against breast (MCF-7) and colorectal (HCT116) cancer cell lines. In addition, molecular docking and in silico pharmacokinetic analyses were performed to support the interpretation of the experimental findings.

## 2. Results and Discussion

### 2.1. Production of Phyllanthus emblica Niosomes

The production process was optimized to yield *Phyllanthus emblica*-loaded niosomes with minimal size and PDI, while exhibiting the highest possible EE.

**Figure 1:**
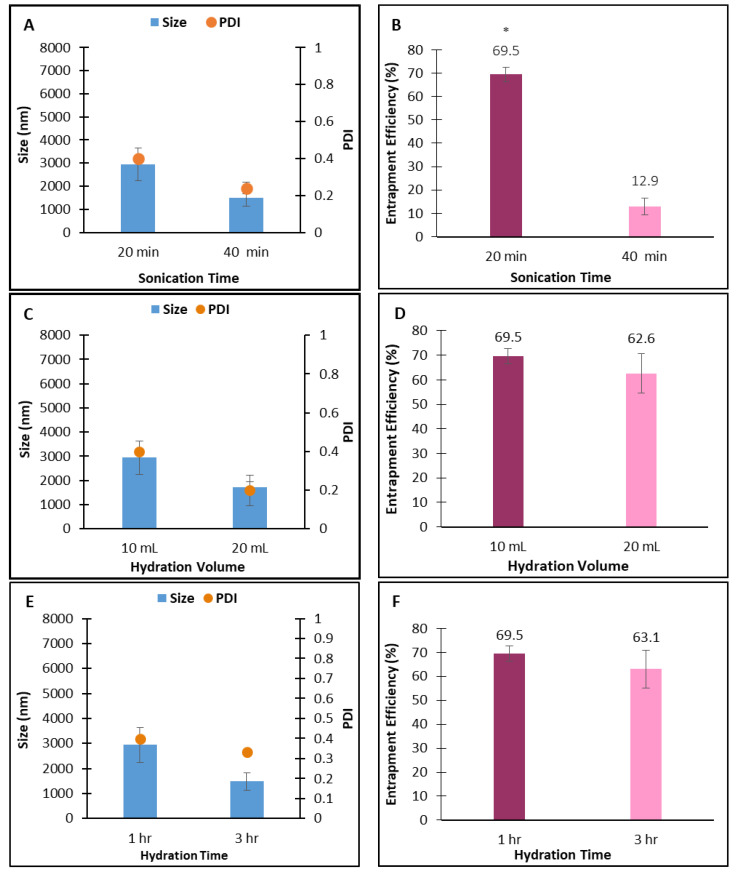

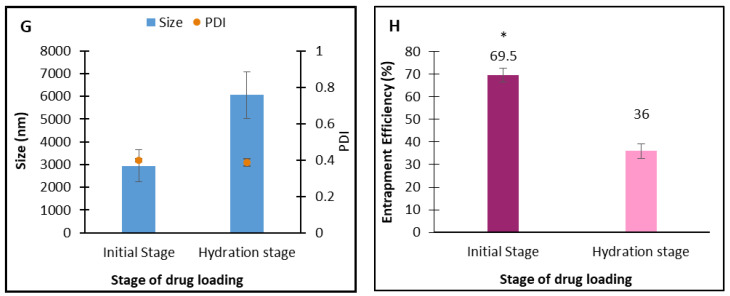
Effect of sonication time on (**A**) size, PDI, and (**B**) %EE; effect of hydration volume on (**C**) size, PDI, and (**D**) %EE; effect of hydration time on (**E**) size, PDI, and (**F**) %EE; effect drug loading stage on (**G**) size, PDI, and (**H**) %EE. The results represent the mean ± SD from 3 independent batches. * indicates statistical significance (*p* < 0.05).

Increasing the sonication time from 20 to 40 min reduced both vesicle size and PDI, from an average of 2943 nm to 1481 nm and from 0.399 to 0.239, respectively (Figure 1A). This effect is likely attributable to the increased energy input during longer sonication, which generates cavitation events and shear forces that fragment larger vesicles into smaller, more uniformly distributed particles. However, the size reduction was statistically insignificant. On the other hand, the %EE was greatly influenced and showed a significant (*p* < 0.05) reduction from an average of 70% to 13% (Figure 1B).

Sonication is a commonly used method of size reduction employed in the production of niosomes [18]. The findings provide insights into the impact of sonication time on particle size uniformity and drug entrapment efficiency and highlight the importance of optimizing the sonication process in niosome production. It is therefore suggested that shorter sonication times are more favorable for achieving optimal drug encapsulation in niosomes. In addition to its size-reducing effect, prolonged sonication may also decrease entrapment efficiency [18]. Several factors could contribute to the decrease in entrapment efficiency with prolonged sonication. These include vesicle disruption and leakage that could result from excessive sonication, potentially leading to loss of the encapsulated amla extract. The intense energy input during extended sonication time could generate higher shear forces, leading to vesicle destabilization and reduced encapsulation efficiency [19]. Furthermore, prolonged sonication can generate heat, potentially promoting drug degradation or oxidation. Heat-sensitive compounds, such as amla extract, could be particularly vulnerable to these effects, thereby reducing entrapment efficiency. It is therefore crucial to maintain a balance between vesicle formation and the potential disruption caused by excessive sonication.

Increasing the hydration volume from 10 mL to 20 mL was accompanied by reductions in vesicle size and PDI (Figure 1C) and %EE (Figure 1D), although these changes were not statistically significant. This indicates that the hydration volume did not significantly influence the measured parameters in this study. It has been reported that reducing the hydration volume can increase %EE to a certain limit, after which the lipid solution may become saturated, leading to a reversal of the effect [7]. Similarly, increasing the hydration time from 1 to 3 h resulted in an insignificant reduction in size and PDI (Figure 1E) and %EE (Figure 1F). Similarly, increasing the hydration time from 1 to 3 h resulted in an insignificant reduction in all measured responses (size, PDI, and %EE). Varying the stage at which the *Phyllanthus emblica* extract was loaded, whether incorporated with the lipids at the initial stage of the formulation or with the buffer at the hydration stage, did not significantly affect the size and PDI of the vesicles, although the average size was smaller upon incorporating the extract at the initial stage (Figure 1G). However, loading the extract at the initial stage resulted in a significantly higher % EE (69.5 ± 5.3%) than when incorporated at the hydration stage (36.0 ± 5.5%; Figure 1H). This suggests that altering the loading method significantly affects the drug’s encapsulation efficiency.

Although 40 min of sonication produced superior niosomes with a smaller size and PDI, the sonication time was set to 20 min due to the significant difference in %EE. A 1 h hydration time was selected because it provided a faster, more efficient production process without significantly affecting the assessed parameters. The optimized parameters selected, therefore, were a sonication time of 20 min, a hydration volume of 10 mL, a hydration time of 1 h, and loading the *emblica* extract at the initial stage.

### 2.2. Physicochemical Characterization of Phyllanthus emblica-Loaded Niosomes

All niosomal formulations were characterized to evaluate their physicochemical properties, and the influence of surfactant type and surfactant-to-cholesterol molar ratio on these properties was studied.

**Figure 2:**
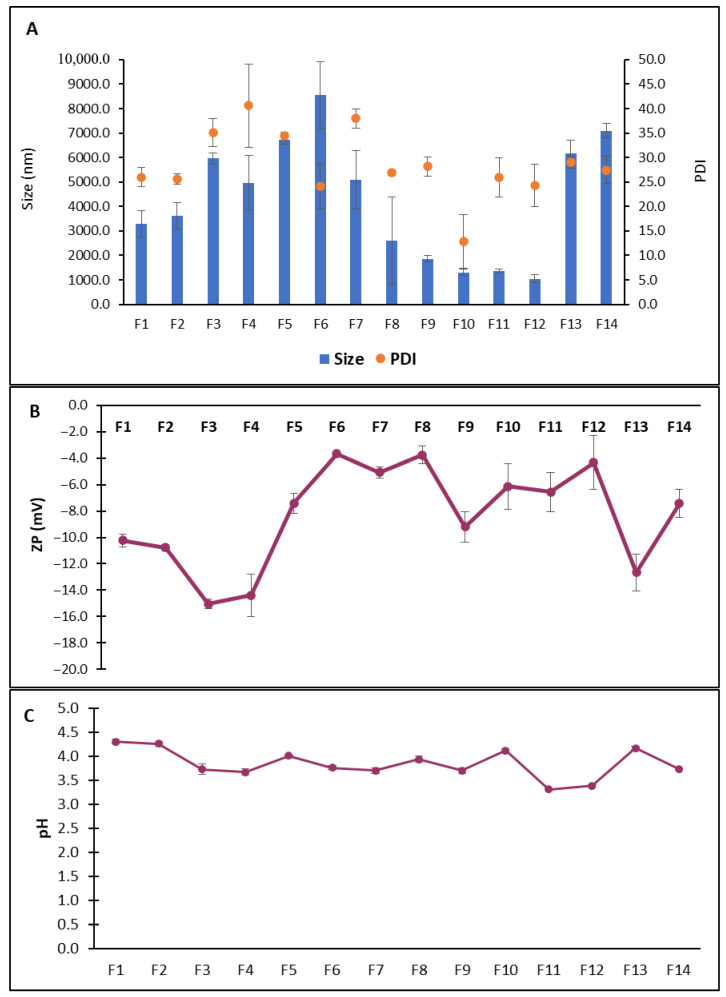
Results of physicochemical characterization: (**A**) Vesicle size and PDI, (**B**) ZP, and (**C**) pH. The results represent the mean ± SD from 3 independent batches.

As represented by Figure 2A, the particle sizes of the formulations ranged from 1042 nm to 8556 nm, indicating the formation of vesicles within micro-size ranges. Formulations containing Brij 35 and Brij 58 (F9–F12) exhibited the smallest particle sizes, with formulations F10–F12 being significantly (*p* < 0.05) smaller in size than other formulations, suggesting that the long hydrophobic chains and moderate ethoxylation of Brij surfactants promote tighter bilayer packing and vesicle curvature, resulting in smaller size [20]. Conversely, Tween 80- and Poloxamer 188-based formulations (F5, F6, F13, F14) produced significantly (*p* < 0.05) larger vesicles (>6 µm). In addition to headgroup steric hindrance, several other factors may contribute to the observed increase in vesicle size. Surfactants with higher hydrophilic–lipophilic balance (HLB) values tend to form more hydrated bilayers, which can promote the formation of larger vesicles. Moreover, the presence of bulky ethoxylated chains in surfactants such as Tween and Poloxamer may introduce steric repulsion between adjacent molecules, limiting tight bilayer packing. Interactions between surfactant molecules and cholesterol may also influence bilayer rigidity and curvature, thereby affecting vesicle formation and size distribution during thin-film hydration [21,22].

The PDI values, which reflect the uniformity of the size distribution, ranged from 0.13 to 0.41, indicating a transition from monodisperse to polydisperse systems. The lowest PDI (*p* < 0.05) was observed for F10, composed of Brij 35 at a 2:1 molar ratio, suggesting that this formulation produced the most uniform vesicle population. In contrast, formulations based on Span 20, Tween 20, and Tween 80 (except for F6 and F8, which had 2:1 molar ratios) showed significantly (*p* < 0.05) higher PDIs (>0.3), indicating heterogeneous vesicle populations [23], possibly due to multilamellar structures or aggregation during formation [24].

The complete DLS measurement output of the optimized formulation and particle size distribution is provided in Supplementary Figure S1. Five independent DLS measurements were performed, three consistent readings were selected and averaged to ensure reliability and minimize the influence of outliers.

All niosomal formulations were negatively charged, with a zeta potential ranging from −3.7 mV to −15.1 mV (Figure 2B). The Span 20 formulations had significantly (*p* < 0.05) lower zeta potential values than other formulations, while the 2:1 formulations of Tween 80, Tween 20, and Brij 58 had a significantly (*p* < 0.05) higher zeta potential, and notably, formulations with higher cholesterol content (1:1) generally had more negative ZP values, which could be attributed to the influence of cholesterol on membrane packing and surface charge distribution. However, all formulations fall below the ±30 mV threshold typically associated with high colloidal stability, suggesting that steric stabilization, contributed by non-ionic surfactants, is likely the main stabilizing mechanism in these systems [25]. Figure 2C shows that all niosomal dispersions exhibited an acidic pH, ranging from 3.3 to 4.3, with minimal variation between formulations, possibly reflecting the use of a common hydration medium. However, the formulations of Span 80 had the highest pH, whereas those of Brij 58 had significantly lower pH (*p* < 0.05).

Overall, the results highlight the importance of surfactant selection and molar ratio optimization in tailoring the physicochemical properties of niosomes.

### 2.3. Determination of Entrapment Efficiency

The content of *Phyllanthus emblica* extract in the formulated niosomes was quantified spectrophotometrically at 276 nm, selected based on the characteristic UV absorption maximum (λ_max_) of its phenolic constituents.

A full UV–Vis spectral scan of the extract (200–800 nm), performed in PBS (pH 7.3), confirmed a distinct absorbance maximum at 276 nm (Supplementary Figure S2). This wavelength was therefore used to estimate total phenolic content during entrapment efficiency determination, in agreement with previous reports that employed UV detection at 270–280 nm for phenolic-rich extracts [26].

**Figure 3:**
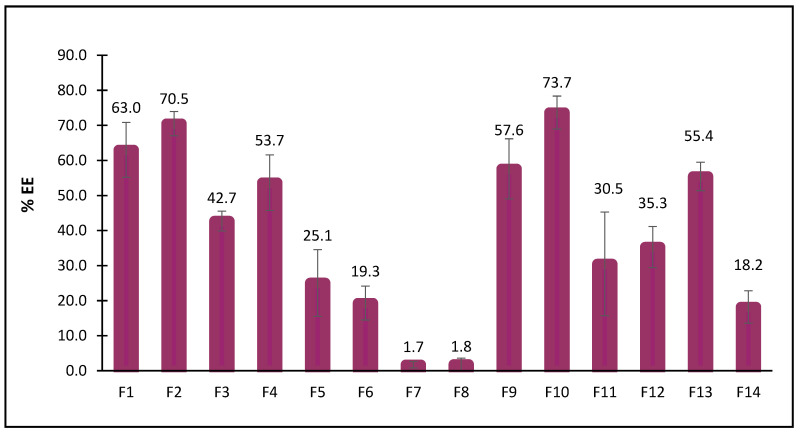
EE% of drug-loaded niosomal formulations prepared with different surfactants at two surfactant-to-cholesterol molar ratios (1:1 and 2:1). Results represent the mean ± SD from 3 independent batches.

The %EE of various formulations varied significantly with the type of surfactant used and the surfactant-to-cholesterol molar ratio (Figure 3).

Formulations F10 and F2, containing Brij 35 at a surfactant to cholesterol molar ratio of 2:1 and Span 80 at a surfactant to cholesterol molar ratio of 2:1, exhibited significantly higher (*p* < 0.05) entrapment efficiencies than all other formulations, with values of 70.5 ± 5.9% and 73.7 ± 8.1%, respectively. These were followed by F1 and F9, which also contained Span 80 and Brij 35, but at a lower surfactant-to-cholesterol molar ratio of 1:1. These results suggest that both Brij 35 and Span 80 are effective in forming stable bilayers capable of encapsulating a substantial amount of drug. The long hydrocarbon chain of Span 80 and the moderate ethoxylation of Brij 35 may enhance bilayer cohesion and membrane rigidity, thereby reducing permeability and improving retention of encapsulated constituents within the vesicles [27,28,29].

In contrast, Tween-based formulations (F5–F8), particularly those containing Tween 20, exhibited very low %EE values; F7 and F8 had significantly lower % EE (*p* < 0.05) than all other formulations, at 1.7% and 1.8%, respectively. This poor performance can be attributed to the high hydrophilicity (high HLB) and bulky polyoxyethylene chains of Tween surfactants, which may increase bilayer permeability and drug leakage during vesicle formation [30].

The Span 20-based formulations (F3, F4) showed a moderate %EE, which was lower than that of the Span 80-based formulations (F1, F2). This is likely due to the longer and more unsaturated alkyl chain of Span 80, which promotes more stable vesicle formation [31]. Similarly, Brij 58-based formulations (F11, F12) exhibited moderate %EE, lower than those achieved with Brij 58 formulations, possibly due to differences in alkyl chain length and headgroup size that affect membrane packing.

In most cases, formulations with a 1:1 ratio exhibited lower %EE than those with a 2:1 ratio. This trend suggests that increasing the surfactant content relative to cholesterol can enhance bilayer flexibility and accommodate more drug molecules [32]. Interestingly, Poloxamer 188-based formulations showed substantial variation in %EE across both surfactant-to-cholesterol molar ratios. F14, with the 2:1 ratio, exhibited a significantly lower (*p* < 0.05) %EE (18.2%) compared to F13, with the 1:1 molar ratio (55.4%). This may be due to Poloxamer’s amphiphilic nature and its limited ability to form rigid bilayers on its own, especially when cholesterol content is reduced [33].

Overall, the results indicate that both surfactant type and surfactant-to-cholesterol ratio significantly influence the entrapment efficiency of amla in niosomal vesicles, underscoring the need to carefully select and optimize formulation components to maximize encapsulation efficiency. Among all formulations, F10 and F2 appeared to be the most promising, exhibiting high loading potential.

### 2.4. In Vitro Release Studies

**Figure 4:**
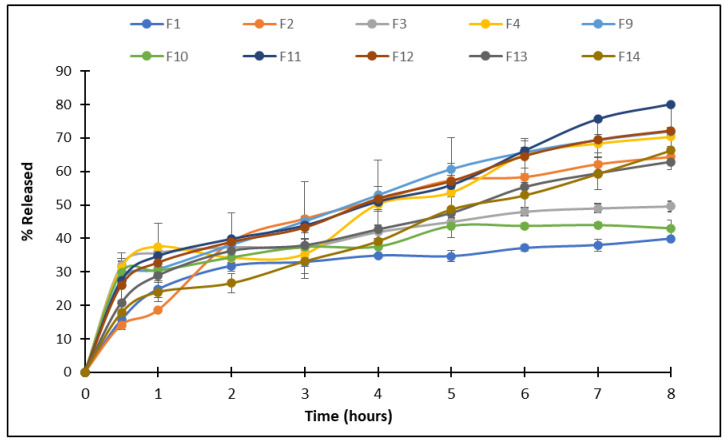
The release profile of *Phyllanthus emblica* from the niosomal formulations incorporating the different surfactants in both surfactant-to-cholesterol molar ratios (1:1 and 2:1). Results represent the mean ± SD from 3 independent batches.

The in vitro release profiles of *Phyllanthus emblica*-loaded niosomal formulations were evaluated over an 8 h period, and the cumulative percentage of drug released at each time point is shown in Figure 4. The results demonstrate that the release behavior varied considerably across the formulations, primarily influenced by the surfactant type and the surfactant-to-cholesterol molar ratio.

Among all formulations, the Brij 58 formulations (F11 and F12) exhibited the highest cumulative release at 8 h, reaching 80.1 ± 0.8% and 72.1 ± 4.1%, respectively. These were closely followed by F9, which contains Brij 35 at a 1:1 surfactant-to-cholesterol molar ratio and reaches a final release of 72.0%. However, F10, with a 2:1 Brij 35 ratio, showed a drop in cumulative release to 43% at 8 h.

In contrast, Span-based formulations showed sustained release, likely due to tighter membrane packing and lower bilayer permeability [8], with the 1:1 formulations showing lower release than the 2:1 formulations (39.9% compared to 64.4% for the Span 80 formulations, and 49.6% compared to 70.3% for the Span 20 formulations), suggesting that increasing the surfactant content improves membrane permeability, thereby promoting higher release [34]. Poloxamer 188-based formulations (F13 and F14) showed moderate release behavior, with cumulative releases of 62.9% and 66.3%, respectively.

These findings highlight that the balance between bilayer rigidity and permeability, modulated by surfactant structure and cholesterol content, is key to achieving controlled drug release. Brij-based formulations offer promising release kinetics for applications requiring rapid drug availability, while Span-based systems may be better suited for sustained-release profiles.

The niosomal formulation F2, composed of Span 80 and cholesterol in a 2:1 ratio, exhibited the highest entrapment efficiency and a more sustained release profile of *Phyllanthus emblica* extract compared to other ratios. This can be attributed to the physicochemical properties of Span 80, a nonionic surfactant with a low hydrophilic–lipophilic balance (HLB ≈ 4.3), which promotes the formation of stable and less permeable bilayers with cholesterol. The presence of cholesterol further enhances membrane rigidity and packing density, thereby reducing vesicular leakage and the diffusion of encapsulated phytoconstituents.

It is important to note that the extract of *Phyllanthus emblica* is a complex mixture of phenolic compounds with varying physicochemical properties, including differences in molecular weight, polarity, and solubility. These differences contribute to the observed variation in release behavior, as lower molecular weight and more hydrophilic constituents (e.g., gallic acid) tend to diffuse more rapidly from the vesicular system, resulting in an initial faster release phase. In contrast, higher molecular weight or less polar compounds (e.g., emblicanin A and B, ellagic acid, and flavonoids) exhibit slower diffusion due to stronger interactions with the lipid bilayer and possible partitioning within the vesicular membrane. This heterogeneous release pattern is characteristic of multi-component herbal extracts and has been widely reported in similar delivery systems, where individual constituents are released at different rates depending on their affinity to the carrier matrix and the surrounding release medium [7,9].

The bioactive compounds of *Phyllanthus emblica*, including hydrolyzable tannins, phenolic acids, and flavonoids, are moderately polar phenolic compounds that absorb in the UV region around 270–280 nm. Monitoring the release at 276 nm, therefore, enabled quantification of the released phenolic fraction. The controlled release observed at this wavelength reflects the gradual diffusion of encapsulated phenolic constituents from the vesicles into the release medium, which may contribute to sustained antioxidant and anticancer activity and improved therapeutic performance.

### 2.5. Release Kinetic Modeling

To closely study the kinetics and mechanism of release of *Phyllanthus emblica* extract from the different formulations, the mean release data were fitted to various kinetic models. Linear regression analysis was performed, and goodness-of-fit was evaluated using the correlation coefficient (R^2^). For each formulation, the model exhibiting the highest R^2^ value was considered the best descriptor of the release profile. The release kinetic models studied were zero-order, first-order, Higuchi, Hixson–Crowell, and Korsmeyer–Peppas. The Korsmeyer–Peppas model was applied to the initial 60% of the cumulative release data, as recommended for mechanistic interpretation. Table 1 presents the correlation coefficients and rate constants obtained for each model, with the highest R^2^ value for each formulation indicated by an asterisk (*).

Among all models, the Higuchi model provided the best overall fit, with the highest R^2^ values for most formulations (Table 1). Since the Higuchi model describes release from a matrix system governed by diffusion, these results indicate that diffusion-controlled release predominates in the niosomal formulations. The Korsmeyer–Peppas model also showed good correlation for several formulations, further supporting the role of diffusional processes. The First-order model demonstrated good correlation for some formulations, suggesting that drug release may be influenced by concentration-dependent mechanisms.

To further compare the in vitro release behavior of the developed niosomal formulations, a model-independent analysis was performed using DDSolver. The calculated parameters, including area under the dissolution curve (AUC), dissolution efficiency (DE), mean dissolution time (MDT), mean residence time (MRT), and relative dispersion (RD), were used to quantitatively describe the extent and time scale of drug release.

The DDSolver results (Table 2) demonstrated that some formulations, particularly Brij-based systems, exhibited higher AUC and DE values, reflecting a greater overall extent of drug release during the study period. However, these parameters primarily describe the magnitude of release rather than the quality or control of the release process. In contrast, the optimized formulation F2 (Span 80:cholesterol, 2:1), although not exhibiting the highest AUC or DE values, showed a more regulated and sustained release pattern, as evidenced by its diffusion-controlled kinetics, absence of burst release, and favorable MDT/MRT values.

Importantly, formulation optimization in controlled drug delivery systems is not solely based on maximizing drug release, but rather on achieving a balance between sustained release, formulation stability, high entrapment efficiency, and therapeutic performance. While DDSolver highlights formulations with higher overall release, the experimental in vitro release profiles, kinetic modeling, and cytotoxicity studies collectively demonstrate that F2 provides a controlled and prolonged release behavior that is more suitable for anticancer applications. Therefore, DDSolver was used as a complementary comparative tool to distinguish between formulations with high release extent and those offering controlled delivery, supporting the rational selection of F2 as the optimized formulation based on overall pharmaceutical and biological performance rather than maximum dissolution alone.

Considering all evaluated parameters, formulation F2 (Span 80: cholesterol, 2:1) was selected as the optimized formulation. F2 demonstrated the highest entrapment efficiency among the tested formulations and exhibited a sustained release profile without pronounced burst release. Although certain Brij-based systems showed higher cumulative release (AUC and DE), their faster release kinetics were less suitable for sustained delivery. In contrast, F2 provided a diffusion-controlled release pattern with favorable MDT and MRT values, supporting its selection for further characterization.

Previous studies have explored the incorporation of *Phyllanthus emblica* extracts into different nanocarrier systems to enhance their biological activity and stability. For example, Iqbal et al. [17] reported the synthesis of *Phyllanthus emblica*-mediated silver nanoparticles that exhibited enhanced antioxidant and anticancer potential due to improved cellular interactions and the bioavailability of the phytochemicals. Although metallic nanoparticle systems have demonstrated promising biological activity, their long-term biocompatibility and stability remain concerns. In addition, vesicular drug delivery systems have been widely investigated for plant-derived bioactive compounds; for instance, Liga et al. [8] described the potential of niosomes as efficient carriers for improving the stability and controlled release of therapeutic agents. In comparison, the niosomal formulation developed in the present study demonstrated favorable physicochemical characteristics, including high entrapment efficiency and sustained diffusion-controlled release, supporting the suitability of nonionic surfactant vesicles for delivering phenolic-rich *Phyllanthus emblica* extracts.

### 2.6. FTIR Spectroscopy Analysis

**Figure 5:**
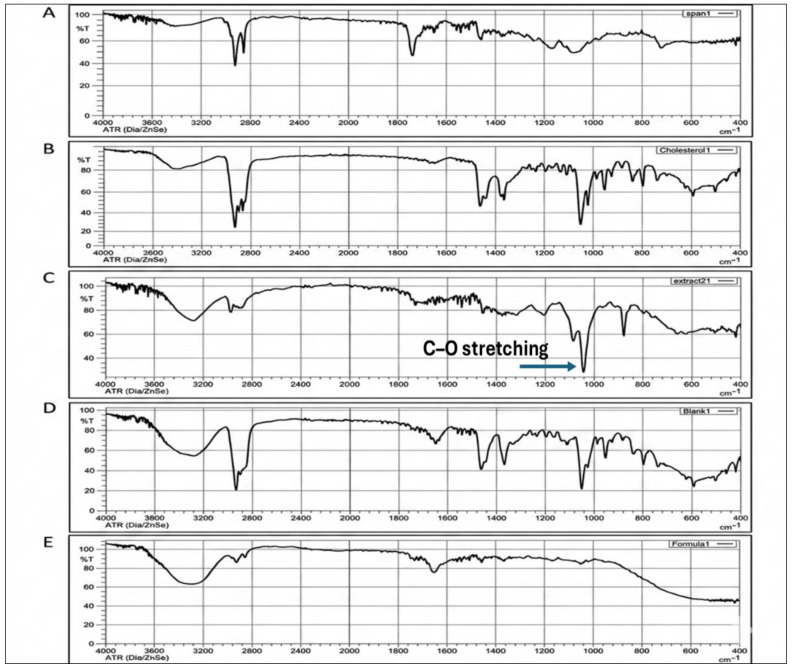
FTIR Spectra of (**A**) Span 80, (**B**) Cholesterol, (**C**) *Phyllanthus emblica* extract, (**D**) Blank optimized formulation, and (**E**) *Phyllanthus emblica* loaded optimized formulation. The spectra were recorded over 4000–400 cm^−1^ to evaluate possible interactions between the extract and the niosomal components. The disappearance of the characteristic C–O stretching band of *Phyllanthus emblica* extract at 1058 cm^−1^ in the niosomal formulation suggests interaction with the surfactant–cholesterol bilayer and successful encapsulation.

The FTIR spectra obtained for the optimized formulation (blank and *Phyllanthus emblica*-loaded), as well as for each of its individual components—Span 80, cholesterol, and amla extract—are presented in Figure 5.

The characteristic C–O stretching band of the *Phyllanthus emblica* extract observed at 1058 cm^−1^ disappeared in the FTIR spectrum of the niosomal formulation. This suggests strong interactions between the phenolic hydroxyl groups of the extract and the surfactant–cholesterol matrix, confirming successful encapsulation of the bioactive constituents within the vesicular structure rather than mere physical mixing.

### 2.7. Cytotoxicity Study

#### 2.7.1. Cell Culture

MCF-7 and HCT116 cells were successfully cultured under the specified aseptic conditions, demonstrating normal adherent growth and typical morphological characteristics. High cell viability was maintained prior to experimental use, and no contamination was observed. The successful maintenance of both cell lines confirmed the suitability of the selected culture media and incubation conditions.

#### 2.7.2. Cell Viability Assay

Given the reported anticancer activity of *Phyllanthus emblica*, this study investigated whether encapsulating the extract in niosomal vesicles could improve its delivery characteristics and enhance its in vitro anticancer activity.

The concentrations reported in the present study (31.25–1000 μg/mL) refer to the total formulation rather than to *Phyllanthus emblica* extract alone. The selected dose range was determined based on previously published cytotoxic studies of *Phyllanthus emblica* extracts [35], while considering the equivalent extract content within the formulation as calculated from the entrapment efficiency, to ensure appropriate interpretation of the cytotoxicity data.

**Figure 6:**
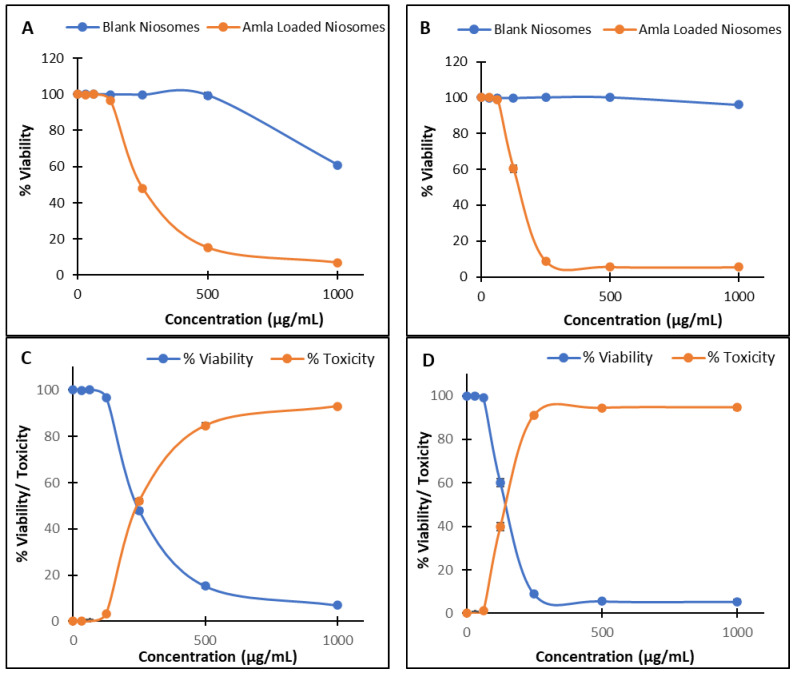
Percentage cell viability of (**A**) MCF-7 cells and (**B**) HCT116 cells, treated with blank formulation (extract-free niosomal formulation) and amla-loaded niosomal formulations at different concentrations (31.25–1000 µg/mL). Concentration-dependent cell viability and corresponding toxicity of amla-loaded niosomal formulation against (**C**) MCF-7 cells and (**D**) HCT116 cells, used to estimate IC_50_.

The cytotoxicity of the *Phyllanthus emblica*-loaded niosomal formulation, and its corresponding blank (drug-free) formulation, was evaluated against MCF-7 (human breast adenocarcinoma) and HCT116 (human colorectal carcinoma) cell lines using the MTT assay. The percentage of viable cells after treatment with increasing concentrations of the formulations (31.25–1000 µg/mL, including 5.2–167.39 μg/mL extract) is presented in Figure 6A,B.

The blank niosomes exhibited negligible cytotoxicity in both cell lines, confirming the carrier system’s biocompatibility. For MCF-7 cells, cell viability remained above 99% across all concentrations except at 1000 µg/mL, where viability decreased to 60.8%. In HCT116 cells, the blank formulation maintained >96% viability even at the highest concentration. These findings indicate that the excipients used (surfactant, cholesterol, etc.) are non-toxic within the tested range and that any subsequent cytotoxic effects observed with the amla-loaded niosomes can be attributed primarily to the encapsulated active compound. In contrast, the drug-loaded formulation showed a strong, concentration-dependent cytotoxicity on both cell lines. At the highest concentration (1000 µg/mL), cell viability dropped drastically to 7.0% in MCF-7 and 5.4% in HCT116 cells, corresponding to 93.0% and 94.6% cytotoxicity, respectively.

A decline in cell viability was observed as formulation concentration increased, particularly above 250 µg/mL, indicating dose-dependent cytotoxicity. Both MCF-7 and HCT116 cells exhibited a dose-dependent reduction in MTT-derived cell viability following treatment with amla-loaded niosomes, compared with the corresponding controls (Figure 6). The decline in cell viability, however, appeared steeper in HCT116 cells, as reflected by lower viability values across most concentrations. The estimated IC_50_ values were 245 µg/mL for MCF-7 cells and 158 µg/mL for HCT116 cells, indicating potent anticancer activity.

In line with our findings demonstrating a clear dose-dependent reduction in cell viability following treatment with *Phyllanthus emblica*-loaded niosomes, previous studies have reported that crude *emblica* preparations exert concentration-dependent antiproliferative effects and induce apoptosis in various human cancer models, including breast and colorectal systems [36]. In contrast, the corresponding blank niosomes in our study exhibited minimal cytotoxicity, confirming the biocompatibility of the carrier system.

These findings support the effective delivery and biological activity of *Phyllanthus emblica* phytoconstituents through the niosomal system. While the present study focused on the development and functional evaluation of niosomal formulation, one limitation is the absence of a direct comparison between the niosomal formulation and the free *Phyllanthus emblica* extract in the in vitro cytotoxicity evaluation. Such a comparison is essential to determine whether niosomal formulation provides a true therapeutic advantage over the free extract. This aspect will be addressed in future studies to further validate the added value of the developed delivery system.

### 2.8. Molecular Docking Studies

Molecular docking analysis was performed to explore the potential interactions between the major phytochemicals identified in the *Phyllanthus emblica* extract and selected cancer-associated molecular targets relevant to breast and colorectal cancers. The binding affinities of the phytochemicals toward estrogen receptor alpha (ERα), β-catenin, PI3Kα, Bcl-2, and cyclooxygenase-2 (COX-2) are summarized in Table 3.

Overall, several flavonoids exhibited strong binding affinities across multiple targets, suggesting a multi-target interaction profile consistent with the polypharmacological nature of plant-derived anticancer agents. Among the tested compounds, rutin and naringin showed the highest binding energies, particularly against COX-2 and PI3Kα, indicating a strong potential to modulate inflammatory and survival pathways associated with tumor progression. Similarly, myricetin, quercetin, kaempferol, and chrysoeriol demonstrated favorable binding toward PI3Kα and β-catenin, key regulators of cancer cell proliferation, apoptosis resistance, and metastasis.

The strong interaction of several phytochemicals with β-catenin is particularly relevant to colorectal cancer, where aberrant activation of Wnt/β-catenin signalling is a hallmark of tumour development and progression. This observation aligns with the higher sensitivity of HCT116 cells observed in the cytotoxicity studies. Likewise, notable binding affinities for ERα and Bcl-2 support the antiproliferative and pro-apoptotic effects observed in MCF-7 breast cancer cells, which rely on estrogenic signalling and anti-apoptotic mechanisms for survival.

Importantly, phenolic acids, such as ellagic acid and caffeic acid, also showed moderate but consistent binding across multiple targets, suggesting that both flavonoid and phenolic constituents contribute to the extract’s anticancer activity. These findings support the concept that the anticancer efficacy of *Phyllanthus emblica* extract arises from synergistic interactions among multiple phytochemicals that act on distinct molecular pathways, rather than from a single dominant compound. Similar multi-target docking profiles have been reported for other flavonoid-rich herbal extracts exhibiting anticancer activity [37,38,39].

Taken together, the docking results provide a molecular-level rationale for the observed in vitro cytotoxicity and support the use of niosomal encapsulation to deliver a complex mixture of bioactive compounds that can simultaneously modulate multiple cancer-related targets.

It should be noted that molecular docking provides predictive insights into possible ligand–target interactions; therefore, the observed binding affinities should be interpreted as supportive computational evidence rather than direct experimental confirmation of the underlying molecular mechanisms.

### 2.9. In Silico Analysis of Pharmacokinetic and Drug-Likeness Predictions

To further assess the translational relevance of the phytochemicals exhibiting the highest docking affinities, in silico pharmacokinetic and drug-likeness predictions were performed using SwissADME. The key predicted parameters for the selected compounds are presented in Table 4.

The analysis revealed that smaller flavonoids such as 7-hydroxyflavone, catechin, quercetin, kaempferol, and chrysoeriol exhibited favorable drug-likeness profiles, with no Lipinski rule violations and moderate predicted bioavailability scores. These compounds also showed high predicted gastrointestinal absorption, supporting their intrinsic potential as bioactive agents. In contrast, larger glycosylated flavonoids such as rutin and naringin displayed low predicted gastrointestinal absorption, high topological polar surface area (TPSA), and multiple Lipinski rule violations, which are commonly associated with limited oral bioavailability.

Despite these limitations, glycosylated flavonoids demonstrated some of the strongest binding affinities in the docking studies, highlighting an important disconnect between pharmacodynamic potential and pharmacokinetic behavior. This discrepancy further justifies the use of a niosomal delivery system. Encapsulation of such poorly permeable but pharmacologically potent compounds within niosomes is expected to enhance their stability, protect them from rapid metabolism, and improve cellular uptake, thereby overcoming inherent biopharmaceutical constraints. Similar strategies have been shown to improve the therapeutic performance of flavonoids and other polyphenolic compounds with poor oral absorption [22,39,40].

Additionally, most of the evaluated phytochemicals showed minimal predicted blood–brain barrier permeability and limited cytochrome P450 inhibition, suggesting a favorable safety profile and reduced risk of off-target central nervous system effects or major drug–drug interactions. These properties are advantageous for anticancer applications where systemic toxicity is a major concern.

Overall, the in silico pharmacokinetic predictions complement the molecular docking and cytotoxicity findings by demonstrating that, although several high-affinity phytochemicals exhibit suboptimal drug-likeness, their incorporation into a niosomal delivery system provides a rational strategy to enhance bioavailability and therapeutic efficacy. This integrated in vitro–in silico approach strengthens the mechanistic basis of the developed *Phyllanthus emblica*-loaded niosomal formulation and supports its potential for further in vivo evaluation.

In summary, our cytotoxicity findings, together with the physicochemical characterization, support the added value of niosomal encapsulation for *Phyllanthus emblica* as an anticancer delivery system.

## 3. Materials and Methods

### 3.1. Materials

Span 80, Tween 20, cholesterol, and Brij 35 were purchased from Alpha Chemika, Mumbai, India. Brij 58 was purchased from Aldrich Chemical Company, Inc., Saint Louis, MO, USA. Tween 80 was purchased from El Nasr Pharmaceutical Chemicals, Kalyoubia, Egypt, and Span 20 from Sigma Aldrich Co. Ltd., Gillingham, UK. 3-(4,5-dimethylthiazol-2-yl)-2,5-diphenyltetrazolium bromide (MTT) was purchased from Bio Basic Inc., Markham, ON, Canada. All the solvents used in this study are of analytical grade. The cell lines were purchased, and the experiment was carried out in the National Cancer Institution, Cairo University, Cairo, Egypt.

### 3.2. Extraction and Purification

*Phyllanthus emblica* fruits were purchased from a local market (Lulu market) in Dubai, UAE, in October 2023. The plant material was identified and authenticated by Prof. Naglaa Shehab, Department of Pharmaceutical Sciences, Dubai Medical University, College of Pharmacy, Dubai, UAE. The fruits (2 kg) were washed, cut, and blended with 50% (*w*/*v*) aqueous alcohol (2 × 2 L). The mixture was then kept in a sonicator at room temperature for seven days. The sonicator temperature was checked regularly during sonication, and whenever a temperature rise was observed, sonication was paused, and cooling was applied until the sample returned to room temperature. The alcoholic portion was removed under reduced pressure at 50 °C using a rotary evaporator, and the remaining water was eliminated by lyophilization. The resulting dried extract was collected and stored in a refrigerator at 4 °C in airtight containers for use in niosome preparation. The phytochemical composition of the *Phyllanthus emblica* extract used in this study has been previously characterized by HPLC analysis, along with the determination of total phenolic and flavonoid contents, as reported by Shehab et al. [6].

### 3.3. Production of Phyllanthus emblica Niosomes

**Figure 7:**
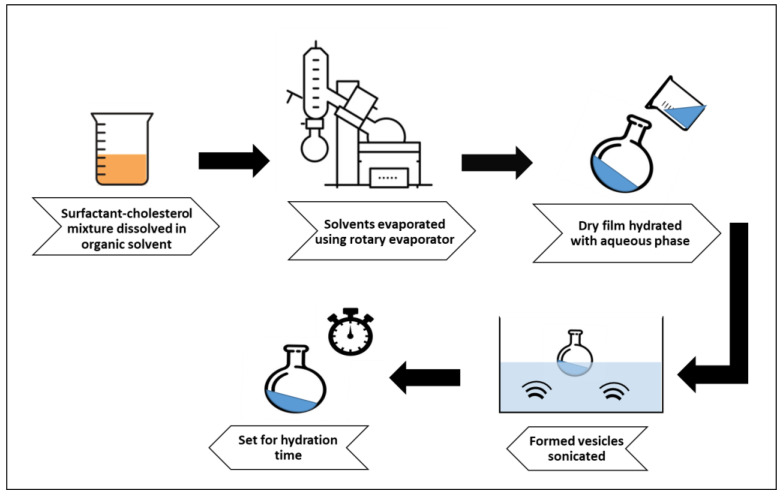
Production steps of *Phyllanthus emblica*-loaded niosomes.

*Phyllanthus emblica* fruit extract-loaded niosomes were prepared by the solvent evaporation-thin-film hydration method [41]. Several formulations were prepared incorporating different surfactants and cholesterol at molar ratios of 1:1 and 2:1, as presented in Table 5. The surfactant-cholesterol mixture was dissolved in 10 mL of a 1:1 chloroform: methanol mixture. The organic solvents were then evaporated using the rotary evaporator (Laborota 4000 efficient, Heidolph, Schwabach, Germany). After evaporation, the dry film formed was hydrated by the aqueous phase of Phosphate-Buffered Saline (PBS), pH 7.3, containing the *Phyllanthus emblica* extract dose. The formulations were then sonicated in a bath sonicator (S60H, Elmasonic, Singen, Germany) to reduce vesicle size and were left to hydrate at room temperature (Figure 7).

The production process was optimized using a systematic optimization methodology implemented through a sequential one-factor-at-a-time (OFAT) approach. Sonication time, hydration volume, hydration time, and the stage of drug loading were adjusted sequentially while keeping other variables constant. Two levels per factor were evaluated (Table 6), and the optimal level was selected based on statistical significance before proceeding to the next parameter. Particle size, polydispersity index (PDI), and entrapment efficiency (EE) were measured as response variables. Statistical analysis was performed using ANOVA in Minitab^®^ 19, with significance assessed at *p* < 0.05.

### 3.4. Determination of Vesicle Size and Zeta Potential

Samples were prepared from each formulation and diluted 1:10 (*v*/*v*) with distilled water to a final volume of 1 mL. The size and PDI of the vesicles were measured by dynamic light scattering (DLS), and values are reported as the intensity-weighted hydrodynamic diameter (Z-average). Zeta potential (ZP) was measured by electrophoretic light scattering (ELS), using the particle analyzer (Litesizer 500, Anton Paar, Graz, Austria). All measurements were made at 25 °C.

### 3.5. Determination of pH

The pH of the niosome formulations was measured using a calibrated digital pH meter (Hanna Instruments, Woonsocket, RI, USA) at room temperature. The instrument was calibrated using standard buffer solutions prior to measurement. Each sample was measured in triplicate, and the average pH value was recorded. The pH assessment is important to evaluate the stability and suitability of the formulations.

### 3.6. Determination of Entrapment Efficiency

The unentrapped extract was separated from the loaded niosomes by centrifugation [41]. A 1 mL sample of niosomal dispersion from each formulation was centrifuged at 15,000 rpm for 30 min at 4 °C using a microrefrigerator (Kubota, Osaka, Japan). The niosomes were precipitated, and the supernatant was collected. UV was used to determine the concentration of the free extract; Vis spectrophotometry (Shimadzu, Kyoto, Japan) at 276 nm was performed using a calibration curve constructed with lyophilized amla extract dissolved in phosphate buffer (pH 7.3) over the concentration range of 10–60 µg/mL, with the same buffer used as the blank. Linear regression analysis yielded the equation “y = 19.941x” with a correlation coefficient (R^2^ = 0.9991). This calibration curve was used to determine the drug content for entrapment efficiency (EE%) calculations and to quantify extract release in the in vitro release study described in the following section. The calibration curve is provided in the Supplementary Material (Supplementary Figure S3).

The percentage entrapment efficiency (% EE) was then determined by Equation (1) [42].

(1) $$\%\;EE=\frac{5\;mg-amount\;of\;free\;extract}{5\;mg}\times 100$$

### 3.7. In Vitro Release Studies

The in vitro release of API from *Phyllanthus emblica*-loaded niosomes was studied using the dialysis method [43]. Dialysis Membranes (14 kDa, Visking, Medicell Membranes Ltd., London, UK) were soaked overnight in the release medium. 3 mL of the niosomal dispersion was placed into a dialysis tube with a 14 kDa molecular weight cutoff, and both ends were sealed. The dialysis bag was then immersed in 300 mL of PBS (pH 7.3), maintained at 37 ± 2 °C, and continuously stirred at 100 rpm. At predetermined time intervals (0.5, 1, 2, 3, 4, 5, 6, 7, and 8 h), samples were withdrawn from the receptor compartment, and an equal volume of fresh medium was replaced to maintain sink conditions. The samples were analyzed spectrophotometrically at 276 nm, and the amount of drug released was determined from the calibration curve of the extract in PBS pH 7.3. The percentage of drug released was determined by Equation (2).

(2) $$\%\;drug\;released=\frac{Amount\;released}{Initial\;amount\;(15\;mg)}\times 100$$

### 3.8. Release Kinetic Modeling

Data obtained from the in vitro release study were fitted to several mathematical models (Table 7). The graph for each model was plotted, and the correlation coefficient (R^2^) was determined. The best fit model was selected based on the highest R^2^, while the mechanism of release was determined from the Korsmeyer model [44].

In addition to kinetic model fitting, a model-independent analysis of the in vitro release data was performed using DDSolver^®^ software, 2010 (Microsoft Excel add-in). The cumulative release data were analyzed to calculate statistical moment parameters, including the area under the dissolution curve (AUC), area between the curve and its asymptote (ABC), mean dissolution time (MDT), mean residence time (MRT), variance of dissolution time (VDT), relative dispersion (RD), and dissolution efficiency (DE). These parameters were used to quantitatively compare the extent and time course of drug release across different niosomal formulations. The DDSolver analysis was employed as a complementary tool to provide an objective comparison of release profiles and to support the interpretation of release behavior alongside kinetic modeling results.

### 3.9. FTIR Spectroscopy Analysis

Fourier Transform Infra-Red (FTIR) spectra were obtained for the optimized formulation as well as for each of its individual components: Span 80, cholesterol, and *Phyllanthus emblica* extract. Samples were analyzed using an FTIR spectrophotometer (Shimadzu, Japan) in the range 400–4000 cm^−1^, following a previously reported protocol [7].

### 3.10. Cytotoxicity Study

#### 3.10.1. Cell Culture

Human breast adenocarcinoma (MCF-7) and human colorectal carcinoma (HCT116) cells were purchased from the National Cancer Institute, Cairo University, Egypt. Both cells were cultured under standard aseptic conditions in a humidified incubator at 37 °C and 5% CO_2_. For MCF-7, cells were maintained in Eagle’s Minimum Essential Medium (EMEM) supplemented with 10% fetal bovine serum (FBS) and 0.01 mg/mL recombinant human insulin. For HCT116, cells were maintained in McCoy’s 5A supplemented with 10% FBS. Where included for routine culture, penicillin–streptomycin (1%) was added [45,46].

#### 3.10.2. Cell Viability Assay

The MTT assay was performed on the MCF7 and HCT116 cell lines as models for human breast cancer and colon cancer, respectively. A 96-well tissue culture plate was inoculated with 1 × 10^5^ cells/mL (100 μL/well) and incubated at 37 °C for 24 h to develop a complete monolayer. Cells (MCF-7 or HCT116) were seeded into designated wells of 96-well plates. Wells allocated to experimental groups (untreated control, vehicle control, and treatment concentrations) were inoculated with cells, whereas blank wells were not inoculated and contained only complete medium. When confluent, the growth medium was decanted, and the cells were washed twice with the wash medium. 0.1 mL of each sample was added to triplicate wells, with control wells receiving only maintenance medium. The plate was incubated at 37 °C and checked for any physical signs of toxicity, e.g., partial or complete monolayer loss, rounding, shrinkage, or cell granulation. 20 μL of MTT solution (5 mg/mL in PBS) was added to each well, and the plate was shaken on a shaking table at 150 rpm for 15 min. The plate was then incubated at 37 °C, 5% CO_2_ for 4 h to allow MTT to be metabolized, after which the media was discarded. The MTT metabolic product (formazan) was re-suspended in 200 μL dimethyl sulfoxide (DMSO), then shaken on a shaking table at 150 rpm for 15 min. The Optical Density (OD) of the samples was measured at 560 nm and correlated to the cell quantity. % viability was determined by Equation (3). The IC_50_ value was determined as the concentration that inhibited cell growth by 50%.

(3) $$\%\;viability=\frac{OD\;of\;sample}{OD\;of\;control}\times 100$$

### 3.11. Molecular Docking Studies

#### 3.11.1. Selection of Receptors

The three-dimensional crystal structures of the receptors were obtained from the RCSB Protein Data Bank (RCSB PDB). Human estrogen receptor alpha ligand-binding domain (PDB ID: 3ERT), PI3Kα (PDB ID: 3ZIM), Bcl-2 (PDB ID: 4MAN), β-catenin (PDB ID: 1JDH), and human cyclooxygenase-2 (COX-2, PDB ID: 5IKR) were selected as potential molecular targets due to their established roles in breast and colorectal cancer progression, survival signaling, inflammation, and apoptosis. These targets are biologically relevant to the MCF-7 breast cancer and HCT116 colorectal cancer cell lines used in the present study.

#### 3.11.2. Protein Preparation

BIOVIA Discovery Studio Visualizer, a free web-based tool (https://www.3ds.com/products/biovia/discovery-studio/visualization, accessed on 12 January 2026), was used to prepare proteins. After the structure was opened in the visualizer, the water molecules and heteroatoms were deleted. Moreover, any bound ligand was deleted from the crystal structure. The next step in preparing the protein was to add polar hydrogen atoms. The file was saved in Protein Data Bank Files (PDB) format. Once the prepared protein was loaded into PyRx^®^ 1.2, we converted it to PDBQT format by treating it as a macromolecule.

#### 3.11.3. Ligand Preparation

The three-dimensional structures of the phytochemicals present in the extract were downloaded from PubChem in the SDF format. Energy minimization of all ligands was performed using the built-in Open Babel tool in PyRx. All structures were then converted to PDBQT format before docking.

#### 3.11.4. Molecular Docking with PyRx

After proteins and ligands were prepared, molecular docking was performed using the Vina Wizard in PyRx. Following the addition of the macromolecule and the library of phytochemicals and reference molecules, the grid box was adjusted to cover the entire protein, known as blind docking.

### 3.12. In Silico Pharmacokinetic and Drug-Likeness Prediction

In silico pharmacokinetic and drug-likeness properties of selected phytochemicals from *Phyllanthus emblica* were predicted using the SwissADME web server (http://www.swissadme.ch, accessed on 12 January 2026). Based on molecular docking results, nine phytochemicals with the highest binding affinities to the selected cancer-associated targets were selected for further analysis. The canonical SMILES of each compound were obtained from the PubChem database and uploaded to SwissADME for evaluation.

The predicted parameters included molecular weight (MW), topological polar surface area (TPSA), gastrointestinal (GI) absorption, blood–brain barrier (BBB) permeability, P-glycoprotein (P-gp) substrate status, cytochrome P450 (CYP3A4) inhibition potential, Lipinski rule-of-five violations, and bioavailability score. These parameters were selected to assess the drug-likeness, biopharmaceutical behaviour, and potential pharmacokinetic limitations of the phytochemicals. The in silico analysis was used to support the rationale for niosomal encapsulation of the extract constituents and to complement the findings from molecular docking and in vitro cytotoxicity.

### 3.13. Statistical Analysis

All experiments were performed in triplicate, and the results were expressed as the mean ± SD. One-way Analysis of variance (ANOVA) with Tukey’s post hoc test was used to determine statistical significance at *p* < 0.05.

## 4. Conclusions

In this study, *Phyllanthus emblica* extract-loaded niosomes were successfully developed and optimized using the thin-film hydration method. Among the investigated formulations, the Span 80–cholesterol system (2:1 molar ratio) showed the most favorable performance, demonstrating high entrapment efficiency, sustained drug release, and diffusion-controlled release behavior consistent with the Higuchi model. FTIR analysis confirmed successful encapsulation of the *Phyllanthus emblica* extract within the niosomal bilayer without evidence of chemical incompatibility. The optimized formulation also showed promising in vitro anticancer activity against MCF-7 and HCT116 cancer cell lines, with clear concentration-dependent cytotoxic effects. In contrast, the corresponding blank formulation showed minimal toxicity, confirming the carrier system’s biocompatibility. These findings support the potential of niosomal encapsulation to effectively deliver phytochemical constituents of *Phyllanthus emblica* and maintain their biological activity. Further studies are warranted to expand on these findings, including a direct comparative evaluation with the free extract to confirm the therapeutic advantage of the niosomal formulation, as well as in vivo investigations, pharmacokinetic profiling, and stability assessment to establish better the therapeutic potential and translational applicability of the developed formulation.

## Figures and Tables

**Table 1:** Correlation coefficient and rate constant of different kinetics models applied to in vitro release data. (*) indicates the highest R^2^ value for each formulation type.

	Zero-Order		First-Order		Higuchi		Hixson–Crowell		Korsmeyer-Peppas		
	R^2^	K_0_ (%.h^−1^)	R^2^	K_1_ (h^−1^)	R^2^	K_H_ (h^−1/2^)	R^2^	K_HC_ (h^−1/3^)	R^2^	K_KP_ (%.h^−1^)	*n*
F1	0.675	3.64	0.732	0.049	0.886	12.8	0.7133	0.068	0.948 *	4.11	0.204
F2	0.869	7.50	0.939	0.126	0.974 *	24.4	0.918	0.163	0.919	3.86	0.557
F3	0.608	4.04	0.711	0.061	0.814	14.3	0.677	0.082	0.876 *	4.61	0.178
F4	0.845	7.04	0.921 *	0.134	0.918	22.5	0.904	0.165	0.759	4.41	0.373
F9	0.887	7.60	0.972	0.146	0.985	24.5	0.951	0.180	0.988 *	4.34	0.435
F10	0.567	3.52	0.645	0.050	0.793	12.8	0.619	0.068	0.936 *	4.40	0.183
F11	0.908	8.14	0.950	0.173	0.968 *	25.8	0.952	0.205	0.917	4.45	0.414
F12	0.891	7.49	0.971	0.143	0.984 *	24.2	0.953	0.177	0.967	4.41	0.402
F13	0.883	6.41	0.953	0.107	0.980 *	20.7	0.935	0.139	0.951	4.21	0.374
F14	0.953	7.12	0.976	0.119	0.976 *	0.813	0.975	0.154	0.940	3.74	0.517

**Table 2:** Model-independent dissolution parameters obtained from DDSolver analysis of the in vitro release profiles of *Phyllanthus emblica*-loaded niosomal formulations, including area under the dissolution curve (AUC), area between the curve and asymptote (ABC), mean dissolution time (MDT), mean residence time (MRT), variance of dissolution time (VDT), relative dispersion (RD), and dissolution efficiency (DE).

Parameter	F1	F2	F3	F4	F9	F10	F11	F12	F13	F14
AUC	255.8	367.5	326.3	385.7	404.8	298.2	412.1	398.6	342.4	319.8
ABC	63.5	147.7	70.5	176.7	171.2	45.9	228.7	178.2	160.8	210.6
MRT	3.77	3.31	3.71	3.31	3.23	3.76	3.16	3.26	3.45	3.39
MDT	1.59	2.29	1.42	2.51	2.38	1.07	2.86	2.47	2.56	3.18
VDT	4.16	3.99	3.88	5.80	5.05	1.98	6.72	5.55	6.12	6.65
m^2^	6.68	9.25	5.90	12.12	10.71	3.11	14.88	11.66	12.65	16.74
RD	1.64	0.76	1.92	0.92	0.89	1.74	0.82	0.91	0.94	0.66
DE	0.32	0.46	0.41	0.48	0.51	0.37	0.52	0.50	0.43	0.40

**Table 3:** Binding energies of Phytochemicals with receptors.

Ligand	3ERT	1JDH	3ZIM	4MAN	5IKR
7-hydroxyflavone	−8.6	−6.6	−8.6	−7.7	−8.6
Rutin	−7.9	−7.8	−9.6	−8.2	−10.1
Naringin	−8.5	−8.3	−9.4	−8.6	−9.9
Myricetin	−7.6	−7.5	−8.6	−7.5	−9.7
Ellagic acid	−6.7	−7	−9.2	−6.9	−9.5
Quercetin	−7.4	−7.2	−8.6	−7.9	−9.2
Catechin	−7.2	−7	−8.1	−7.2	−9.2
Chrysoeriol	−7.9	−6.5	−9.4	−7.1	−9
Kaempferol	−7.8	−6.5	−8.2	−7.1	−8.9
Caffeic acid	−6.4	−5.2	−7	−6.2	−7
Gallic acid	−5.8	−5.1	−6.1	−5.6	−6.5
Protocatechuic acid	−5.7	−5.3	−6.3	−5.5	−6.2
Eugenol	−5.9	−5.4	−6.2	−5.8	−6.1
Syringic acid	−5.4	−5	−5.9	−5.8	−6.1
Salicylic acid	−5.5	−5.1	−5.9	−5.4	−6.1

**Table 4:** In silico pharmacokinetic and drug-likeness properties of selected high-affinity phytochemicals from *Phyllanthus emblica*.

Compound	MW (g/mol)	TPSA (Å^2^)	GI Absorption	BBB Permeant	P-gp Substrate	CYP3A4 Inhibitor	Lipinski Violations	Bioavailability Score
7-Hydroxyflavone	238.24	50.44	High	Yes	No	Yes	0	0.55
Catechin	290.27	110.38	High	No	Yes	No	0	0.55
Chrysoeriol	300.26	100.13	High	No	No	Yes	0	0.55
Ellagic acid	302.19	141.34	High	No	No	No	0	0.55
Kaempferol	286.24	111.13	High	No	No	Yes	0	0.55
Quercetin	302.24	131.36	High	No	No	Yes	0	0.55
Myricetin	318.24	151.59	Low	No	No	Yes	1	0.55
Naringin	580.53	225.06	Low	No	Yes	No	3	0.17
Rutin	610.52	269.43	Low	No	Yes	No	3	0.17

**Table 5:** Niosomal formulations incorporating different surfactants and cholesterol.

Formula Code	Surfactant	Surfactant: Cholesterol (Molar Ratio)	Amount of Surfactant (g)	Amount of Cholesterol (g)
F1	Span 80	1:1	0.0857	0.0773
F2	Span 80	2:1	0.1714	0.0773
F3	Span 20	1:1	0.0693	0.0773
F4	Span 20	2:1	0.1386	0.0773
F5	Tween 80	1:1	0.2620	0.0773
F6	Tween 80	2:1	0.5240	0.0773
F7	Tween 20	1:1	0.2456	0.0773
F8	Tween 20	2:1	0.4912	0.0773
F9	Brij 35	1:1	0.2399	0.0773
F10	Brij 35	2:1	0.4798	0.0773
F11	Brij 58	1:1	0.2248	0.0773
F12	Brij 58	2:1	0.4496	0.0773
F13	Poloxamer 188	1:1	0.1680	0.0773
F14	Poloxamer 188	2:1	0.3360	0.0773

Dose of *Phyllanthus emblica* extract is 50 mg.

**Table 6:** Optimization process parameters and their levels.

Factor	Levels	Values
Sonication Time	2	20 min, 40 min
Hydration Volume	2	10 mL, 20 mL
Hydration Time	2	1 h, 3 h
Stage of Drug Loading	2	Initial-stage, Hydration-stage

**Table 7:** Kinetic models applied to the in vitro release data of amla niosomes.

Kinetic Model	Equation Used
Zero order [34]	Q_t_ = Q_0_ + K_0_t, where Q_t_ is the amount of drug released at time t, Q_0_ is the initial amount of drug in the formulation, and K_0_ is the zero-order release constant.
First order [34]	Ln C_t_ = Ln C_0_ − K_t_, where C_t_ is the amount of drug remaining at time t, C_0_ is the initial amount of drug, and K is the first-order release constant.
Higuchi [34]	Q = K_H_ t^1/2^, where Q is the amount of drug released at time t and KH is the Higuchi release constant.
Hixson–Crowell [34]	C_0_^1/3^ − C_t_^1/3^ = K_HCt_, where C_0_ is the initial amount of drug, C_t_ is the amount of drug remaining at time t, and KHC is the Hixson–Crowell release constant.
Korsmeyer-Peppas [44]	M_t_/M_∞_ = K t^n^, where M_t_/M_∞_ is the fraction of drug released at time t, K is the release constant, and n is the release exponent indicating the mechanism of drug release.

## Data Availability

The original contributions presented in this study are included in the article. Further inquiries can be directed to the corresponding authors.

## Supplementary Materials

The following supporting information can be downloaded at https://www.mdpi.com/article/10.3390/ph19040582/s1, Figure S1. Raw dynamic light scattering (DLS) measurement of the optimized niosomal formulation, showing particle size distribution (intensity-based) and associated measurement parameters obtained using a Litesizer 500 instrument. Five independent measurements were performed; three consistent readings were selected and averaged to ensure reliability and reduce the impact of outliers. Figure S2. UV–Vis absorption spectrum of *Phyllanthus emblica* extract (200–800 nm) in PBS (pH 7.3), showing a maximum absorbance (λmax) at 276 nm, used for spectrophotometric determination of total phenolic content. Figure S3. Calibration curve of *Phyllanthus emblica* extract in phosphate buffer (pH 7.3) over the concentration range of 10–60 µg/mL, obtained by UV–Vis spectrophotometry at 276 nm, showing linear regression (y = 19.941x, R^2^ = 0.9991) used for quantification of total phenolic content.
