# Supplementary material for: Development of Phyllanthus emblica Extract-Loaded Niosomes for Cancer Treatment: Formulation and In Vitro Evaluation
Source: Pharmaceuticals (Basel). 2026 Apr 6;19(4):582. doi: 10.3390/ph19040582 (PMC13118586; doi:10.3390/ph19040582)
Supplement: Supplementary file 1 [file pharmaceuticals-19-00582-s001.zip › pharmaceuticals-4157833-supplementary.pdf]

“Compiled Supplementary Information”

a) Reading 1

Particle size distribution (intensity)

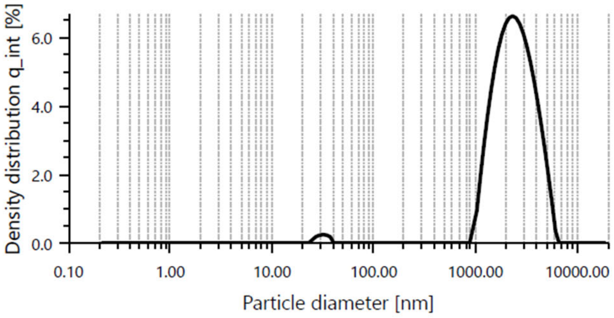

Result

**Hydrodynamic diameter**	3623 nm	**Mean intensity**	223.3 kcounts/s
**Polydispersity index**	24.0 %	**Absolute intensity**	747625.1 kcounts/s
**Diffusion coefficient**	0.1 μm²/s	**Intercept g1²**	0.5360
**Transmittance**	2.7 %	**Baseline**	1.177

Particle size distribution peaks (intensity)

Peak name	Size [nm]	Area	Standard deviation [nm]
Peak 1	2625	98.93 %	988.6
Peak 2	31.96	1.07 %	3.46
Peak 3	-	-	-

b) Reading 2

Particle size distribution (intensity)

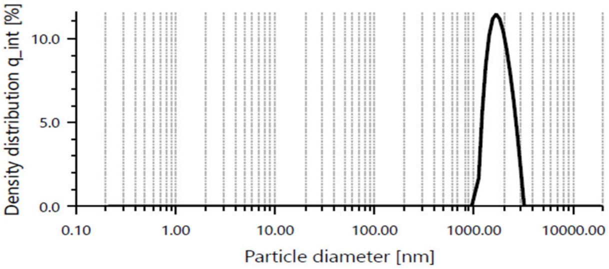

Result

**Hydrodynamic diameter**	2663 nm	**Mean intensity**	197.9 kcounts/s
**Polydispersity index**	27.6 %	**Absolute intensity**	346233.3 kcounts/s
**Diffusion coefficient**	0.2 μm²/s	**Intercept g1²**	0.6347
**Transmittance**	0.3 %	**Baseline**	1.051

Particle size distribution peaks (intensity)

Peak name	Size [nm]	Area	Standard deviation [nm]
Peak 1	1843.3	100.00 %	408.9
Peak 2	-	-	-
Peak 3	-	-	-

c) Reading 3

Particle size distribution (intensity)

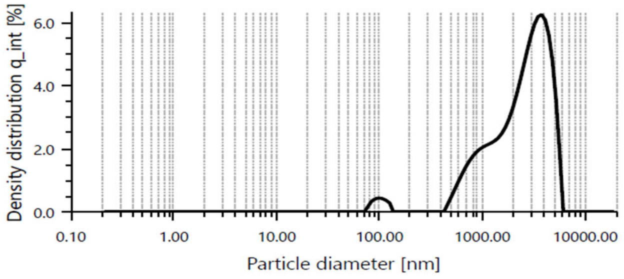

Result

**Hydrodynamic diameter**	4537 nm	**Mean intensity**	408.5 kcounts/s
**Polydispersity index**	35.1 %	**Absolute intensity**	280048.5 kcounts/s
**Diffusion coefficient**	0.1 µm²/s	**Intercept g1²**	0.5645
**Transmittance**	20.3 %	**Baseline**	1.100

Particle size distribution peaks (intensity)

Peak name	Size [nm]	Area	Standard deviation [nm]
Peak 1	2747	97.76 %	2431
Peak 2	102.83	2.24 %	12.88
Peak 3	-	-	-

d) Reading 4

Particle size distribution (intensity)

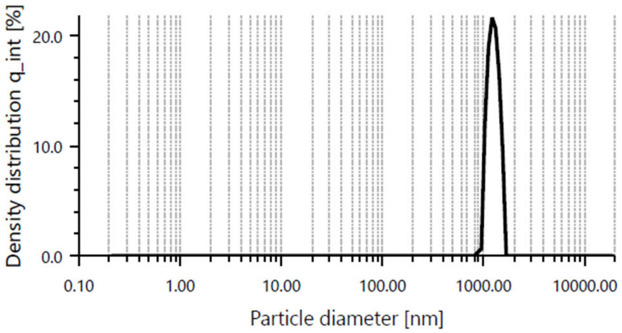

Result

**Hydrodynamic diameter**	1892.5 nm	**Mean intensity**	199.0 kcounts/s
**Polydispersity index**	33.1 %	**Absolute intensity**	153562.3 kcounts/s
**Diffusion coefficient**	0.3 µm²/s	**Intercept g1²**	0.6245
**Transmittance**	4.7 %	**Baseline**	1.241

Particle size distribution peaks (intensity)

Peak name	Size [nm]	Area	Standard deviation [nm]
Peak 1	1262.8	100.00 %	150.14
Peak 2	-	-	-
Peak 3	-	-	-

e) Reading 5

Particle size distribution (intensity)

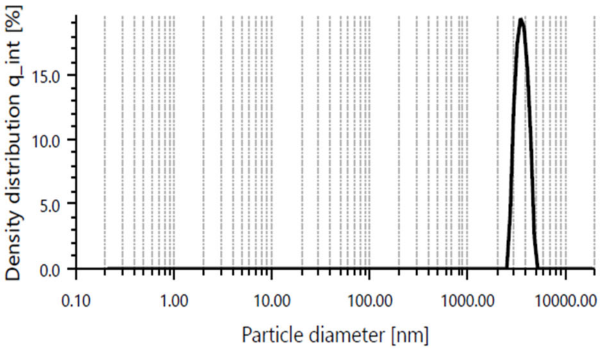

Result

**Hydrodynamic diameter**	5026 nm	**Mean intensity**	314.5 kcounts/s
**Polydispersity index**	25.1 %	**Absolute intensity**	110159.9 kcounts/s
**Diffusion coefficient**	0.1 µm²/s	**Intercept g1²**	0.6466
**Transmittance**	0.4 %	**Baseline**	1.041

Particle size distribution peaks (intensity)

**Peak name**	**Size [nm]**	**Area**	**Standard deviation [nm]**
Peak 1	3620	100.00 %	493.4
Peak 2	-	-	-
Peak 3	-	-	-

Figure S1. Raw dynamic light scattering (DLS) measurement of the optimized niosomal formulation, showing particle size distribution (intensity-based) and associated measurement parameters obtained using a Litesizer 500 instrument. Five independent measurements were performed; three consistent readings were selected and averaged to ensure reliability and reduce the impact of outliers.

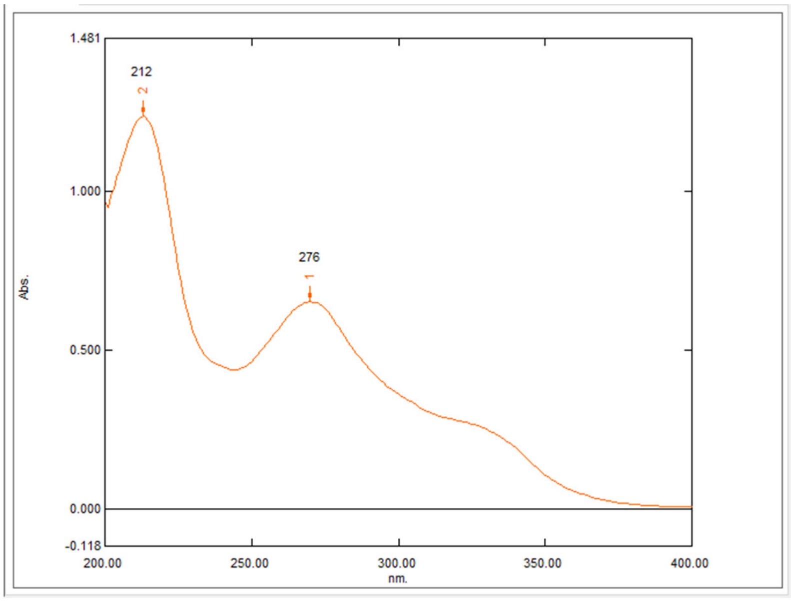

Figure S2. UV–Vis absorption spectrum of *Phyllanthus emblica* extract (200–800 nm) in PBS (pH 7.3), showing a maximum absorbance (λmax) at 276 nm, used for spectrophotometric determination of total phenolic content.

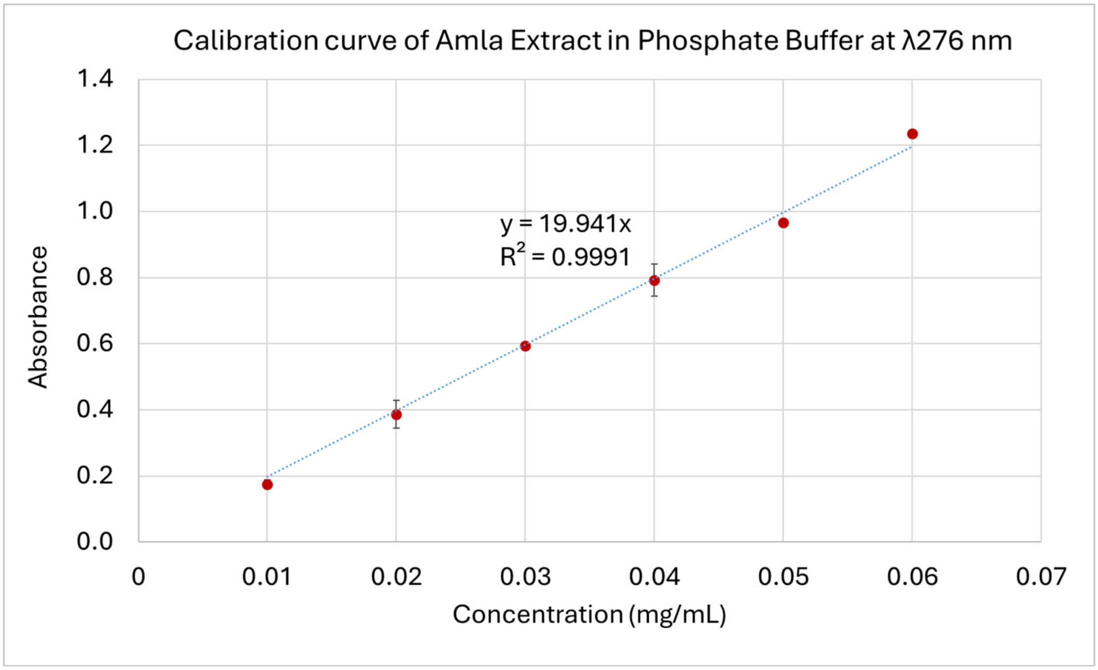

Figure S3. Calibration curve of *Phyllanthus emblica* extract in phosphate buffer (pH 7.3) over the concentration range of 10–60 µg/mL, obtained by UV–Vis spectrophotometry at 276 nm, showing linear regression ($y = 19.941x$, $R^2 = 0.9991$) used for quantification of total phenolic content.
